# Charlson comorbidity index, neutrophil-to-lymphocyte ratio and undertreatment with renin-angiotensin-aldosterone system inhibitors predict in-hospital mortality of hospitalized COVID-19 patients during the omicron dominant period

**DOI:** 10.3389/fimmu.2022.958418

**Published:** 2022-08-25

**Authors:** Andrea Sonaglioni, Michele Lombardo, Adriana Albini, Douglas M. Noonan, Margherita Re, Roberto Cassandro, Davide Elia, Antonella Caminati, Gian Luigi Nicolosi, Sergio Harari

**Affiliations:** ^1^ Division of Cardiology, Istituto di Ricovero e Cura a Carattere Scientifico (IRCCS) MultiMedica, Milan, Italy; ^2^ European Institute of Oncology (IEO) Istituto di Ricovero e Cura a Carattere Scientifico (IRCCS), Milan, Italy; ^3^ Immunology and General Pathology Laboratory, Department of Biotechnology and Life Sciences, University of Insubria, Varese, Italy; ^4^ Unit of Molecular Pathology, Immunology and Biochemistry, Istituto di Ricovero e Cura a Carattere Scientifico (IRCCS) MultiMedica, Milan, Italy; ^5^ Division of Internal Medicine, Istituto di Ricovero e Cura a Carattere Scientifico (IRCCS) MultiMedica, Milan, Italy; ^6^ Division of Pneumology, Semi Intensive Care Unit, Istituto di Ricovero e Cura a Carattere Scientifico (IRCCS) MultiMedica, Milan, Italy; ^7^ Division of Cardiology, Policlinico San Giorgio, Pordenone, Italy; ^8^ Department of Clinical Sciences and Community Health, Università Di Milano, Milan, Italy

**Keywords:** COVID-19, Charlson comobidity index, neutrophil-to-lymphocyte ratio, angiotensin-converting enzyme inhibitors/angiotensin II receptor blockers, mortality

## Abstract

**Purpose:**

To investigate the clinical predictors of in-hospital mortality in hospitalized patients with Coronavirus disease 2019 (COVID-19) infection during the Omicron period.

**Methods:**

All consecutive hospitalized laboratory‐confirmed COVID-19 patients between January and May 2022 were retrospectively analyzed. All patients underwent accurate physical, laboratory, radiographic and echocardiographic examination. Primary endpoint was in-hospital mortality.

**Results:**

74 consecutive COVID-19 patients (80.0 ± 12.6 yrs, 45.9% males) were included. Patients who died during hospitalization (27%) and those who were discharged alive (73%) were separately analyzed. Compared to patients discharged alive, those who died were significantly older, with higher comorbidity burden and greater prevalence of laboratory, radiographic and echographic signs of pulmonary and systemic congestion. Charlson comorbidity index (CCI) (OR 1.76, 95%CI 1.07-2.92), neutrophil-to-lymphocyte ratio (NLR) (OR 1.24, 95%CI 1.10-1.39) and absence of angiotensin-converting enzyme inhibitors (ACEI)/angiotensin II receptor blockers (ARBs) therapy (OR 0.01, 95%CI 0.00-0.22) independently predicted the primary endpoint. CCI ≥7 and NLR ≥9 were the best cut-off values for predicting mortality. The mortality risk for patients with CCI ≥7, NLR ≥9 and not in ACEI/ARBs therapy was high (86%); for patients with CCI <7, NLR ≥9, with (16.6%) or without (25%) ACEI/ARBs therapy was intermediate; for patients with CCI <7, NLR <9 and in ACEI/ARBs therapy was of 0%.

**Conclusions:**

High comorbidity burden, high levels of NLR and the undertreatment with ACEI/ARBs were the main prognostic indicators of in-hospital mortality. The risk stratification of COVID-19 patients at hospital admission would help the clinicians to take care of the high-risk patients and reduce the mortality.

## Introduction

The novel B.1.1.529 severe acute respiratory syndrome coronavirus-2 (SARS-CoV-2) variant was first detected in South Africa and was named Omicron by WHO on Nov 26, 2021 (1).

This variant has many mutations in the spike gene, which may reduce the effectiveness of available vaccines and antibody therapeutics (2).

Due to the variant’s increased transmissibility (3) and ability to evade immunity conferred by previous infection or vaccination (4), a rapid increase in SARS-CoV-2 infections was observed in all WHO regions (5), and at the beginning of 2022 Omicron accounted for more than 89% of sequenced samples globally (6).

With the pandemic still growing worldwide and with the limited healthcare capacity, early prediction of COVID-19 severity and mortality is crucial for improving management and treatment of infected patients (7).

Population studies (8) suggest that the risk of severe outcomes following infection with Omicron might be lower than that observed for previous variants such as Delta, and this risk is attenuated further in those who have received a booster vaccination (9).

However, the total number of hospital admissions and deaths due to Omicron might still be substantial, depending on the role exerted by age and comorbidities in influencing disease severity.

As far as we know, data on outcomes following Omicron infection in older populations with high rates of comorbidity are scanty.

Given the large number of elderly patients with multiple comorbidities who were referred to the Pneumology Division of our Institution during the last few months, we hypothesized that clinical factors as the number of comorbidities, the inflammatory status and the current medical treatment could have contributed to different outcomes.

Accordingly, the present study was primarily designed to investigate the main independent predictors of in-hospital mortality in a retrospective cohort of COVID-19 patients admitted to the Pneumology Division during the Omicron dominant period.

## Methods

### Study population

All consecutive COVID-19 patients who were admitted to the Pneumology Division of the MultiMedica IRCCS (Milano, Italy) from January 1 to May 15, 2022 (the Omicron dominant period), entered this retrospective observational study.

The inclusion criteria were: 1) confirmed SARS-CoV-2 infection by reverse-transcriptase polymerase chain reaction (RT-PCR) assays on material collected by a nasopharyngeal and oropharyngeal swab; 2) patients who were hospitalized; 3) patients who underwent chest X-rays (CXR) on the day of hospital admission.

Patients with negative results for SARS-CoV-2 infection by RT-PCR, patients who died on admission, patients without baseline data or transferred to other designated hospitals during hospitalization were excluded from the analysis.

Following patients’ characteristics were collected from the medical records: age; gender; body surface area (BSA); body mass index (BMI); information about COVID-19 vaccination (subjects vaccinated with 3 doses, with 2 doses, with 1 dose or unvaccinated, respectively); relevant cardiovascular risk factors (hypertension, type 2 diabetes mellitus, smoking, dyslipidemia); electrocardiographic (ECG) data (cardiac rhythm and heart rate); main comorbidities, such as chronic obstructive pulmonary disease (COPD), history of coronary artery disease (CAD), previous stroke/transient ischemic attack (TIA), peripheral vascular disease, chronic kidney disease (CKD), cancer, chronic cognitive deficit; blood tests comprehensive of complete blood count for determining hemoglobin concentration, white blood cells (WBCs) count and neutrophil-to-lymphocyte ratio (NLR), serum levels of creatinine and estimated glomerular filtration rate (eGFR) (10), serum levels of C-reactive protein (CRP), procalcitonin, D-dimer, high-sensitivity (HS) troponine I and N-terminal pro-B-type natriuretic peptide (NT-proBNP); the medical treatment at hospital admission and the COVID-19 in-hospital treatment; finally, the length of hospitalization or days until hospital death.

**Figure 1:**
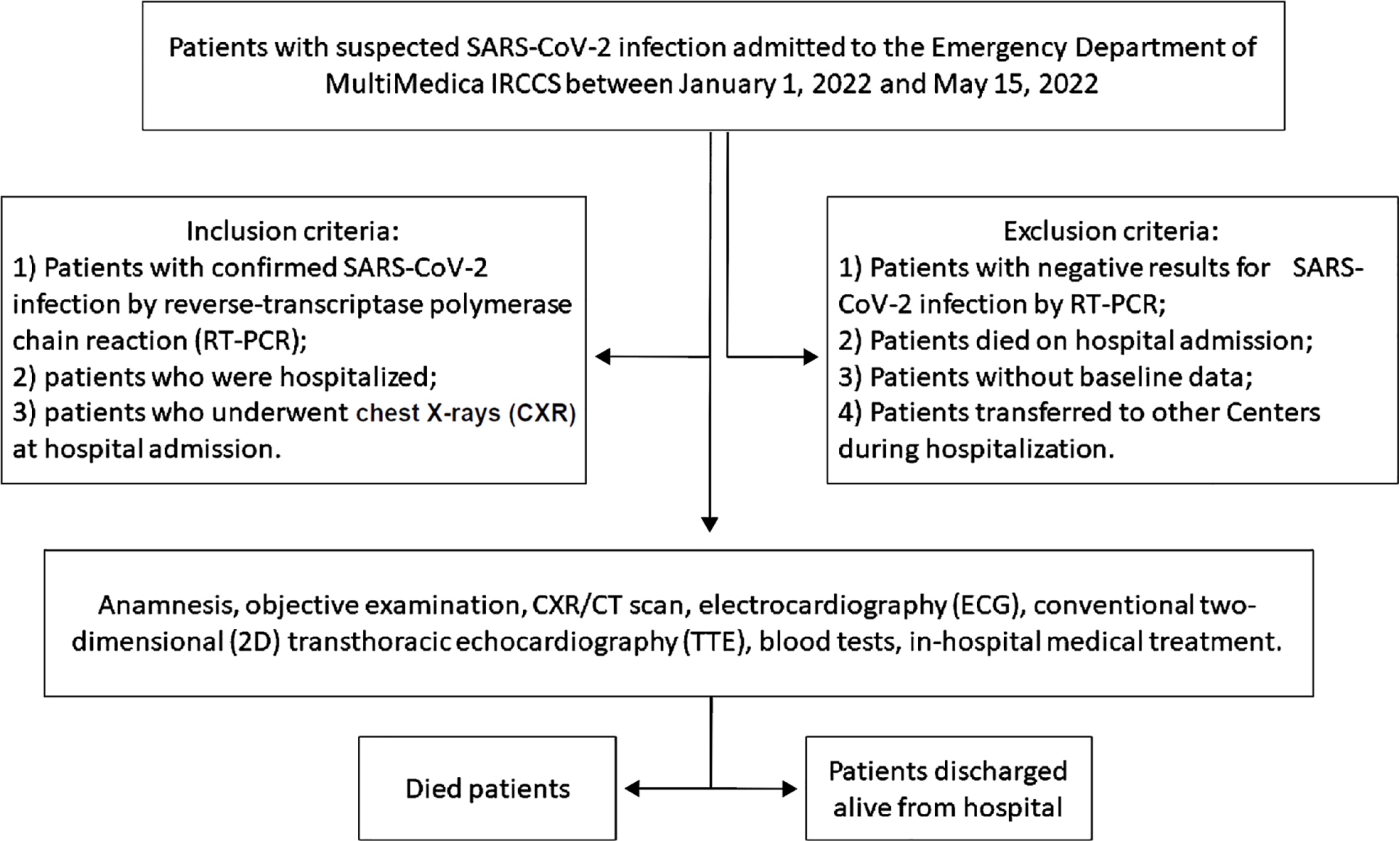
The study design flowchart. 2D, two-dimensional; CXR, chest X-rays; CT, computed tomography; ECG, electrocardiography; RT-PCR, reverse-transcriptase polymerase chain reaction; TTE, transthoracic echocardiography.

All hospitalized COVID-19 patients included in the present study underwent accurate anamnesis, objective examination, CXR and/or CT scan, ECG and conventional two-dimensional (2D) transthoracic echocardiography (TTE). COVID-19 patients who died during the hospitalization and those who were discharged alive were separately analyzed. The study design flowchart is depicted in **Figure 1**.

All procedures were performed according to the ethical standards of the institutional research committee and to the Declaration of Helsinki (1964) and its subsequent amendments or equivalent ethical standards. The study protocol was authorized by the local Ethics Committee (Committee′s reference number 436.2020) and the need for informed consent was not required due to the retrospective nature of the study.

### Comorbidity assessment

To assess the comorbidity burden, the Charlson comorbidity index (CCI) was retrospectively calculated for each COVID-19 patient. The CCI assigned 1 point for each of the following comorbidities: previous or actual myocardial infarction, history of congestive heart failure, peripheral vascular disease, dementia, cerebrovascular disease, chronic lung disease, connective tissue disease, ulcer, chronic liver disease, diabetes; 2 points for each of hemiplegia, moderate or severe kidney disease, diabetes with end-organ damage, tumor, leukemia, lymphoma; 3 points for moderate or severe liver disease; and 6 points for tumor metastasis or AIDS (11).

### Radiographic examinations

Radiology data were collected from the Radiology department of our Institution. All COVID-19 patients underwent CXR at hospital admission, and were evaluated for the presence of unilateral or bilateral pneumonia, pulmonary hilar congestion, unilateral or bilateral pleural effusion, or for the absence of pulmonary alterations (negative examination). Computed tomography (CT) pulmonary angiography was performed only in patients with clinical or laboratory suspicion of pulmonary embolism complicating COVID-19 pneumonia. In selected cases, high resolution computed tomography (HRCT) was also performed.

### Conventional transthoracic echocardiography and lung ultrasound

Echocardiograhic examinations were performed by two sonographers and by an expert cardiologist (AS) by using Philips Sparq ultrasound machine (Philips, Andover, Massachusetts, USA) with a 2.5 MHz transducer.

Following 2D echocardiographic parameters were retrospectively recorded: left ventricular ejection fraction (LVEF) estimated with the biplane modified Simpson’s method (12); average E/e’ ratio, as index of left ventricular diastolic function (13); systolic pulmonary artery pressure (SPAP), derived by the modified Bernoulli equation, where SPAP = 4 x (tricuspid regurgitation velocity)^2^ + right atrial pressure (14). The latter was estimated from inferior vena cava diameter and collapsibility.

Finally, the presence of multiple B-lines, which are the sonographic sign of lung interstitial syndrome (15), was researched from the anterior, lateral and posterior chest, by using Philips Sparq ultrasound machine (Philips, Andover, Massachusetts, USA) with a 12-4 MHz linear transducer. A number of three or more B lines in any given region was considered a pathological finding.

### Primary endpoint

The present study was primarily designed to identify the independent predictors of “in-hospital mortality” in a retrospective cohort of COVID-19 patients.

Details concerning the causes of death of COVID-19 patients were determined by accessing medical records available in the hospital archive and/or from telephone interviews.

### Statistical analysis

To calculate the sample size of COVID-19 patients included in the present study, we hypothesized that COVID-19 patients with higher comorbidity burden (as expressed by CCI) might have a significantly increased risk of “in-hospital mortality” than those with lower comorbidity burden. Statistical power analysis revealed that a sample size of 20 COVID-19 patients who died in hospital and 54 COVID-19 patients discharged alive from hospital reached 80% of statistical power to detect a 3 points difference in the CCI between the two groups of patients with a standard deviation (SD) of 3.0 for each parameter, using a two-sided equal-variance t-test with a level of significance (alpha) of 5%.

For the whole cohort of COVID-19 patients and for the two groups of dead and alive patients, continuous data were summarized as mean ± SD, while categorical data were presented as number (%).

The correlation between CCI and NLR in the whole study population was assessed by Spearman Correlation Coefficient.

Univariate logistic regression analysis was performed to evaluate the effect of the main demographic, clinical, biochemical, and instrumental variables, on the occurrence of the primary endpoint, in our cohort of COVID-19 patients. For each variable investigated, correspondent odds ratios with 95% confidence intervals (CIs) were calculated. Only the variables with statistically significant association on univariate analysis (p value <0.05) were thereafter included in the multivariate logistic regression model.

The receiver operating characteristics (ROC) curve analysis was performed to establish the sensitivity and the specificity of the continuous variables that resulted independently associated with the above-mentioned endpoint. Area under curve (AUC) was estimated. The optimal cutoff of these predictors was calculated using the maximum value of the Youden Index (determined as sensitivity + [1-specificity]).

Statistical analysis was performed with SPSS version 26 (SPSS Inc., Chicago, Illinois, USA), with two-tailed p values below 0.05 deemed statistically significant.

## Results

Between January 1 and May 15, 2022, a total of 74 consecutive laboratory‐confirmed COVID-19 patients (mean age 80.0 ± 12.6 yrs, 45.9% males) were retrospectively analyzed.

Twenty COVID-19 patients (27% of total) died during the hospitalization, whereas the remaining 54 patients (73% of total) were discharged alive.

**Table 1** summarizes baseline clinical characteristics of the whole study population and of the two groups of COVID-19 patients.

**Table 1:** Baseline clinical characteristics of the whole study population and of the two groups of COVID-19 patients.

	All patients (n = 74)	Dead (n = 20)	Alive (n = 54)	P value
**Demographics and anthropometrics**				
Age (yrs)	80.0 ± 12.6	85.1 ± 10.6	78.1 ± 13.1	**0.03**
Male sex (%)	34 (45.9)	14 (70.0)	20 (37.0)	**0.01**
BSA (m^2^)	1.79 ± 0.25	1.74 ± 0.24	1.80 ± 0.24	0.34
BMI (Kg/m^2^)	24.5 ± 4.8	23.7 ± 5.6	24.8 ± 4.5	0.38
**Anti-COVID-19 vaccination**				
Vaccination with 3 doses of COVID-19 vaccine (%)	26 (35.1)	2 (10.0)	24 (44.4)	**0.006**
Vaccination with 2 doses of COVID-19 vaccine (%)	20 (27.0)	8 (40.0)	12 (22.2)	0.13
Vaccination with 1 dose of COVID-19 vaccine (%)	11 (14.9)	2 (10.0)	9 (16.7)	0.47
Unvaccinated (%)	17 (23.0)	8 (40.0)	9 (16.7)	**0.03**
**Cardiovascular risk factors**				
Hypertension (%)	46 (62.2)	15 (75.0)	31 (57.4)	0.16
Type 2 diabetes mellitus (%)	22 (29.7)	4 (20.0)	18 (33.3)	0.26
Current or ex-smokers (%)	21 (28.4)	7 (35.0)	14 (25.9)	0.44
Dyslipidemia (%)	21 (28.4)	5 (25.0)	16 (29.6)	0.69
Obesity (%)	11 (14.9)	3 (15.0)	8 (14.8)	0.98
**Relevant comorbidities**				
COPD (%)	20 (27.0)	6 (30.0)	14 (25.9)	0.72
History of CAD (%)	16 (21.6)	9 (45.0)	7 (13.0)	**0.003**
Previous stroke/TIA (%)	6 (8.1)	3 (15.0)	3 (5.5)	0.19
Peripheral vascular disease (%)	22 (29.7)	10 (50.0)	12 (22.2)	**0.02**
CKD (%)	33 (44.6)	13 (65.0)	20 (37.0)	**0.03**
Cancer (%)	16 (21.6)	6 (30.0)	10 (18.5)	0.29
Chronic cognitive deficit (%)	18 (24.3)	7 (35.0)	11 (20.4)	0.19
CCI	7.4 ± 3.1	9.8 ± 2.7	6.5 ± 2.8	**<0.001**
**Medical treatment at hospital admission**				
Antiplatelets (%)	20 (27.0)	7 (35.0)	13 (24.1)	0.35
Anticoagulants (%)	16 (21.6)	5 (25.0)	11 (20.4)	0.67
Beta blockers (%)	29 (39.2)	4 (20.0)	25 (46.3)	**0.03**
ACE-i/ARBs (%)	33 (44.6)	1 (5.0)	32 (61.1)	**<0.001**
Calcium channel blockers (%)	15 (20.3)	4 (20.0)	11 (20.4)	0.97
Diuretics (%)	18 (24.3)	5 (25.0)	13 (24.1)	0.93
Statins (%)	21 (28.4)	2 (10.0)	19 (35.2)	**0.03**
Oral antidiabetics (%)	11 (14.9)	3 (15.0)	8 (14.8)	0.98
Insulin (%)	11 (14.9)	1 (5.0)	10 (18.5)	0.15

ACEI, angiotensin-converting-enzyme inhibitors; ARBs, angiotensin receptor blockers; BMI, body mass index; BSA, body surface area; CAD, coronary artery disease; CCI, Charlson comorbidity index; CKD, chronic kidney disease; COPD, chronic obstructive pulmonary disease; COVID-19, Coronavirus disease 2019; TIA, transient ischemic attack.

Significant P values are in bold.

Overall, our series of hospitalized COVID-19 patients had advanced age, normal BMI (24.5 ± 4.8 Kg/m^2^), mild-to-moderate prevalence of the most common cardiovascular risk factors and high comorbidity burden, as assessed by CCI (7.4 ± 3.1). Approximately one-third of COVID-19 patients (35.1%) completed the vaccination cycle, 27% of total received 2 doses of COVID-19 vaccine, 14,9% of total received 1 dose of COVID-19 vaccine and the remaining 23% were unvaccinated. As expected, the prevalence of unvaccinated subjects was significantly higher among dead patients in comparison to those discharged alive (40.0 vs 16.7%, p = 0.03).

Compared to COVID-19 patients discharged alive from hospital, those who died in hospital were significantly older (85.1 ± 10.6 vs 78.1 ± 13.1 yrs, p = 0.03) and with a predominance of males (70.0 vs 37.0%, p = 0.01). Distribution of the common cardiovascular risk factors was similar in the two groups of patients. Analysis of comorbidities revealed that patients who died had a significantly greater comorbidity burden than those discharged alive (CCI 9.8 ± 2.7 vs 6.5 ± 2.8, p <0.001). Notably, dead patients showed a significantly increased prevalence of history of CAD, peripheral vascular disease and chronic kidney disease.

Concerning medical treatment at hospital admission, a general underprescription of cardioprotective drugs was observed in COVID-19 patients. Indeed, less than half of patients were regularly treated with beta blockers (39.2%) and angiotensin-converting-enzyme inhibitors (ACEI)/angiotensin receptor blockers (ARBs) (44.6%), and less than one third of patients received antiplatelets (27%), anticoagulants (21.6%), calcium channel blockers (20.3%) and statins (28.4%). The underprescription of cardioprotective drugs was particularly evident among patients who died. Only 5%, 10% and 20% of them did regular use of ACEI/ARBs, statins and beta blockers respectively, at hospital admission.

Symptoms and signs at hospital admission, biochemical parameters, main instrumental findings, and finally details regarding the in-hospital medical treatment of COVID-19 infection, are listed in **Table 2**.

**Table 2:** Symptoms and signs at hospital admission, blood tests, radiographic, ECG and echographic data, and details concerning the in-hospital treatment of COVID-19 infection detected in the whole study population and in the two groups of COVID-19 patients.

	All patients (n = 74)	Dead (n = 20)	Alive (n = 54)	P value
**Symptoms and physical examination at hospital admission**				
Dry cough (%)	35 (47.3)	10 (50.0)	25 (46.3)	0.77
Dyspnea (%)	47 (63.5)	17 (85.0)	30 (55.5)	**0.02**
No symptoms (%)	18 (24.3)	1 (5.0)	17 (31.5)	**0.01**
BT >37.3°C (%)	34 (45.9)	10 (50.0)	24 (44.4)	0.67
SBP (mmHg)	125.9 ± 20.7	127.9 ± 17.9	125.1 ± 21.7	0.60
DBP (mmHg)	74.8 ± 10.3	76.1 ± 8.8	74.3 ± 10.9	0.51
**Blood tests**				
Hb (g/dl)	12.8 ± 2.2	13.3 ± 2.4	12.6 ± 1.8	0.18
WBCs (× 10^9^/L)	10.5 ± 5.2	12.6 ± 5.9	9.7 ± 4.7	**0.03**
NLR	11.5 ± 11.9	23.6 ± 14.8	7.0 ± 6.6	**<0.001**
CRP (mg/dl)	8.6 ± 7.3	10.1 ± 9.5	8.0 ± 6.4	0.28
Procalcitonin (ng/ml)	0.97 ± 2.42	1.13 ± 1.03	0.9 ± 2.8	0.72
Creatinine	1.26 ± 1.00	1.78 ± 1.14	1.07 ± 0.87	**0.006**
eGFR (ml/min/m^2^)	63.7 ± 30.7	48.0 ± 34.9	69.6 ± 27.0	**0.006**
HS troponine I (ng/L)	31.3 ± 75.3	52.3 ± 63.8	23.5 ± 78.2	0.14
D-dimer (ng/ml)	3522.6 ± 6054.8	3661.0 ± 8161.1	3471.4 ± 5158.3	0.90
NT-proBNP (pg/ml)	1605.7 ± 3211.9	2915.7 ± 4356.6	1120.6 ± 2553.0	**0.03**
**Radiographic findings on CXR/CT scan**				
Unilateral pneumonia (%)	12 (16.2)	4 (20.0)	8 (14.8)	0.59
Bilateral pneumonia (%)	28 (37.8)	8 (40.0)	20 (37.0)	0.81
Pulmonary hilar congestion (%)	10 (13.5)	7 (35.0)	3 (5.5)	**0.001**
Unilateral pleural effusion (%)	6 (8.1)	4 (20.0)	2 (3.7)	**0.02**
Bilateral pleural effusion (%)	6 (8.1)	4 (20.0)	2 (3.7)	**0.02**
Pneumonia + PE (%)	3 (4.0)	1 (5.0)	2 (3.7)	0.80
Negative CXR/CT scan (%)	18 (24.3)	1 (5.0)	17 (31.5)	**0.01**
**ECG data**				
Heart rate (bpm)	85.2 ± 19.3	93.4 ± 20.7	82.2 ± 18.1	**0.02**
AF (%)	11 (14.9)	8 (40.0)	3 (5.5)	**<0.001**
**Main echographic variables**				
LVEF (%)	52.5 ± 12.3	41.7 ± 14.0	56.5 ± 8.8	**<0.001**
Average E/e’ ratio	13.4 ± 5.2	15.9 ± 5.0	12.6 ± 5.1	**0.02**
SPAP (mmHg)	39.5 ± 10.6	47.7 ± 13.2	36.5 ± 7.7	**<0.001**
≥3 B-lines on lung ultrasound	21 (28.4)	10 (50.0)	11 (20.4)	**0.01**
**COVID-19 in-hospital treatment**				
No oxygen therapy (%)	25 (33.8)	1 (5.0)	24 (44.4)	**0.001**
Low-flow oxygen therapy (%)	25 (33.8)	7 (35.0)	18 (33.3)	0.89
High-flow oxygen therapy (%)	24 (32.4)	12 (60.0)	12 (22.2)	**0.002**
Subcutaneous enoxaparin (%)	61 (82.4)	19 (95.0)	42 (77.7)	0.08
Intravenous dexamethasone (%)	56 (75.7)	19 (95.0)	37 (68.5)	**0.02**
Intravenous antibiotics (%)	56 (75.7)	19 (95.0)	37 (68.5)	**0.02**
Intravenous diuretics (%)	50 (67.6)	18 (90.0)	32 (59.2)	**0.01**
Intravenous remdesivir (%)	3 (4.0)	0 (0.0)	3 (5.5)	0.56
**Length of hospital stay (days)**	12.1 ± 9.3	10.5 ± 6.2	12.7 ± 10.3	0.37

AF, atrial fibrillation; BT, body temperature; CRP, C-reactive protein; CT, computed tomography; CXR, chest X-rays; COVID-19, Coronavirus disease 2019; DBP, diastolic blood pressure; ECG, electrocardiographic; eGFR, estimated glomerular filtration rate; Hb, hemoglobin; HS, high-sensitivity; LVEF, left ventricular ejection fraction; NLR, neutrophil-to-lymphocyte ratio; NT-proBNP, N-terminal pro-B-type natriuretic peptide; SBP, systolic blood pressure; SPAP, systolic pulmonary artery pressure; WBCs, whilte blood cells.

Significant P values are in bold.

Main symptoms detected in COVID-19 patients at hospital admission were dyspnea (63.5%) and dry cough (47.3%); 45.9% of patients had fever. The prevalence of asymptomatic patients was significantly greater among those patients who were discharged alive (31.5 vs 5.0%, p = 0.01), whereas the dyspnea was much more commonly observed among those patients who died during hospitalization (85.0 vs 55.5%, p = 0.02). Blood pressure values were similar in the two groups of COVID-19 patients and only two cases of arterial hypotension (systolic blood pressure <100 mmHg) were reported.

As regards blood tests results, our study group was found with a significant increase in serum levels of inflammatory biomarkers, as WBCs, NLR, CRP, procalcitonin, with a mild chronic renal failure, and finally with a marked increase in serum levels of D-dimer and NT-proBNP. In comparison to COVID-19 patients who were discharged alive, those who died had significantly higher serum levels of WBCs (12.6 ± 5.9 vs 9.7 ± 4.7 × 10^9^/L, p = 0.03), NLR (23.6 ± 14.8 vs 7.0 ± 6.6, p <0.001) and NT-proBNP (2915.7 ± 4356.6 vs 1120.6 ± 2553.0 pg/ml, p = 0.03) and significantly impaired renal function (eGFR 48.0 ± 34.9 vs 69.6 ± 27.0 ml/min/m^2^, p = 0.006). On the other hand, serum levels of CRP, procalcitonin, HS troponin and D-dimer were similar in the two groups of patients.

On CXR/CT scan, 37.8% of the whole study population was diagnosed with bilateral pneumonia, whereas an acute pulmonary embolism was diagnosed in only 4% of COVID-19 patients, probably due to an extensive prophylactic anticoagulation regimen. The prevalence of bilateral and/or unilateral pneumonia did not differ between dead and alive COVID-19 patients. The latter were more frequently diagnosed with unilateral and/or bilateral pleural effusion. Radiological examinations were totally normal in approximately one-third of alive COVID-19 patients.

The prevalence of atrial fibrillation on ECG was 18.9% of the entire cohort of patients, without statistically significant difference between the two groups of patients (30 vs 14.8%, p = 0.14). However, Group 1 patients had significantly higher heart rate (93.4 ± 20.7 vs 82.2 ± 18.1 bpm, p = 0.02) than Group 2 patients.

On 2D-TTE, LVEF (52.5 ± 12.3%) was substantially preserved in the entire study group and a mild increase in left ventricular filling pressures (LVFP), expressed by the average E/e’ ratio (13.4 ± 5.2), and SPAP (39.5 ± 10.6 mmHg) was observed. COVID-19 patients who died during the hospitalization were diagnosed with significantly lower LVEF (41.7 ± 14.0 vs 56.5 ± 8.8%, p <0.001), significantly higher average E/e’ ratio (15.9 ± 5.0 vs 12.6 ± 5.1, p = 0.02) and significantly increased SPAP (47.7 ± 13.2 vs 36.5 ± 7.7 mmHg, p <0.001), in comparison to COVID-19 patients discharged alive. On lung ultrasound, three or more B-lines were detected in 28.4% of the whole study group, with significantly increased prevalence in patients who died in comparison to those who were discharged alive (50 vs 20.4%, p = 0.01).

Concerning COVID-19 in-hospital treatment, great majority of patients were treated with subcutaneous enoxaparin (82.4%), intravenous dexamethasone (75.7%), intravenous antibiotics (75.7%) and intravenous diuretics (67.6%). Those patients who died were more commonly treated with high-flow oxygen therapy (60.0 vs 22.2%, p = 0.002), intravenous dexamethasone (95.0 vs 68.5%, p = 0.02), intravenous antibiotics (95.0 vs 68.5%, p = 0.02) and intravenous diuretics (90.0 vs 59.2%, p = 0.01) than those who were discharged alive.

Finally, the length of hospital stay was not significantly different in the two groups of patients (10.5 ± 6.2 vs 12.7 ± 10.3 days, p = 0.37).

**Figure 2:**
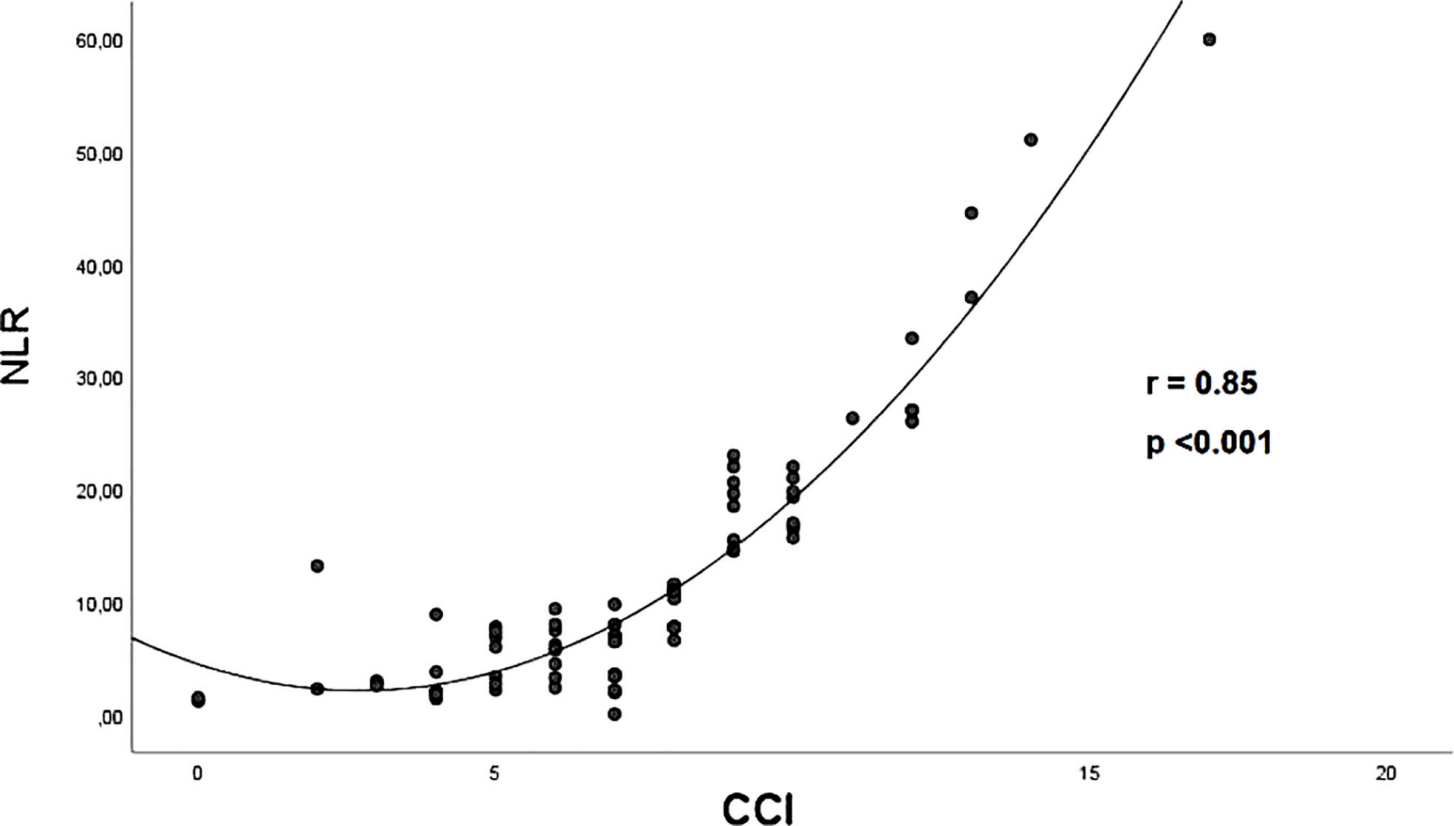
The correlation between CCI score and NLR in the whole study population, assessed by Spearman Correlation Coefficient. CCI, Charlson comorbidity index; NLR, neutrophil-to-lymphocyte ratio.

**Figure 2** illustrates the strong correlation between CCI score and NLR (r = 0.85) observed in the whole study population.

On univariate logistic regression analysis (**Table 3**), following variables were independently correlated with the primary endpoint “in-hospital mortality”: vaccination with 3 doses (OR 0.14, 95%CI 0.03-0.66, p = 0.01); CCI (OR 1.57, 95%CI 1-22-2.03, p <0.001); NLR (OR 1.19, 95%CI 1.09-1.29, p <0.001); eGFR (OR 0.97, 95%CI 0.95-0.99, p = 0.009); LVEF (OR 0.89, 95%CI 0.84-0.94, p <0.001); SPAP (OR 1.11, 95%CI 1.05-1.18, p <0.001); ACEI/ARBs therapy (OR 0.03, 95%CI 0.01-0.27, p = 0.001); finally, high-flow oxygen therapy (OR 3.56, 95%CI 1.22-10.4, p = 0.02).

**Table 3:** Univariate and multivariate logistic regression analysis performed for identifying the main independent predictors of in-hospital mortality in our cohort of hospitalized COVID-19 patients.

VARIABLES	UNIVARIATE LOGISTIC REGRESSION ANALYSIS			MULTIVARIATE LOGISTIC REGRESSION ANALYSIS		
	OR	95% CI	P value	OR	95% CI	P value
**Demographics**						
Age (yrs)	1.05	0.99-1.11	0.08			
Male sex	2.32	0.80-6.73	0.12			
**Anti-COVID-19 vaccination**						
Vaccination with 3 doses	0.14	0.03-0.66	**0.01**	0.25	0.01-4.42	0.34
**Cardiovascular risk factors**						
Hypertension	2.22	0.71-7.01	0.17			
Type 2 diabetes mellitus	1.08	0.36-3.17	0.89			
Obesity	1.01	0.24-4.28	0.98			
Smoking	1.54	0.51-4.63	0.44			
Dyslipidemia	1.05	0.32-3.45	0.93			
**Clinical comorbidity index**						
CCI	1.57	1-22-2.03	**<0.001**	1.76	1.07-2.92	**0.02**
**Blood tests**						
NLR	1.19	1.09-1.29	**<0.001**	1.24	1.10-1.39	**0.001**
CRP (mg/dl)	1.04	0.97-1.11	0.29			
Procalcitonin (ng/ml)	1.04	0.85-1.26	0.72			
eGFR (ml/min/m^2^)	0.97	0.95-0.99	**0.009**	0.98	0.95-1.03	0.46
HS troponine I (ng/L)	1.00	0.99-1.01	0.19			
D-dimer (ng/ml)	1.00	0.92-1.09	0.90			
NT-proBNP (pg/ml)	1.00	0.87-1.15	0.95			
**Instrumental findings**						
Bilateral pneumonia on CXR/CT scan	1.13	0.39-3.24	0.82			
AF	1.22	0.39-3.80	0.72			
LVEF (%)	0.89	0.84-0.94	**<0.001**	0.93	0.80-1.07	0.31
Average E/e’ ratio	1.07	0.96-1.19	0.24			
SPAP (mmHg)	1.11	1.05-1.18	**<0.001**	1.07	0.92-1.24	0.36
**Medical treatment at hospital admission**						
Antiplatelets	1.69	0.56-5.16	0.35			
Anticoagulants	1.31	0.39-4.37	0.67			
Beta blockers	0.58	0.19-1.73	0.33			
ACEi-ARBs	0.03	0.01-0.27	**0.001**	0.01	0.00-0.22	**0.006**
Statins	0.79	0.25-2.55	0.69			
**In-hospital treatment of COVID-19 infection**						
High-flow oxygen therapy	3.56	1.22-10.4	**0.02**	2.28	0.69-7.50	0.17
Subcutaneous enoxaparin (%)	1.14	0.32-4.07	0.84			
Intravenous dexamethasone (%)	1.38	0.43-4.41	0.59			
Intravenous antibiotics (%)	1.84	0.53-6.33	0.33			
Intravenous diuretics (%)	1.60	0.53-4.82	0.40			

ACEI, angiotensin-converting-enzyme inhibitors; AF, atrial fibrillation; ARBs, angiotensin receptor blockers; CCI, Charlson comorbidity index; COVID-19, Coronavirus disease 2019; CRP, C-reactive protein; CT, computed tomography; CXR, chest X-rays; eGFR, estimated glomerular filtration rate; HS, high-sensitivity; LVEF, left ventricular ejection fraction; NLR, neutrophil-to-lymphocyte ratio; NT-proBNP, N-terminal pro-B-type natriuretic peptide; SPAP, systolic pulmonary artery pressure.

Significant P values are in bold.

On multivariate logistic regression analysis (**Table 3**), CCI (OR 1.76, 95%CI 1.07-2.92, p = 0.02) and NLR (OR 1.24, 95%CI 1.10-1.39, p = 0.001) were linearly correlated with the outcome “in-hospital mortality”, whereas ACEI/ARBs therapy (OR 0.01, 95%CI 0.00-0.22, p = 0.006) showed a strong inverse correlation with the primary endpoint.

**Figure 3:**
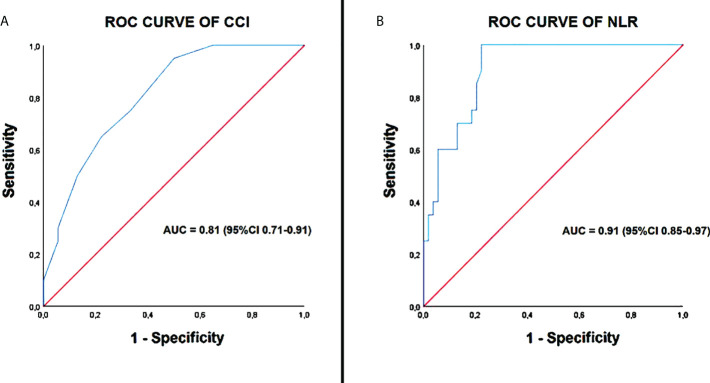
ROC curve analysis of CCI **(A)** and NLR **(B)**. CCI, Charlson comorbidity index; NLR, neutrophil-to-lymphocyte ratio; ROC, receiver operating characteristic curve.

ROC curve analysis highlighted following cut-off values for CCI (≥7; 95% sensitivity and 67% specificity; AUC = 0.81) and NLR (≥9; 100% sensitivity and 78% specificity; AUC = 0.91), as the cut-off values with the best sensitivity and specificity for predicting the outcome “in-hospital mortality” in our study population (**Figure 3**).

**Figure 4:**
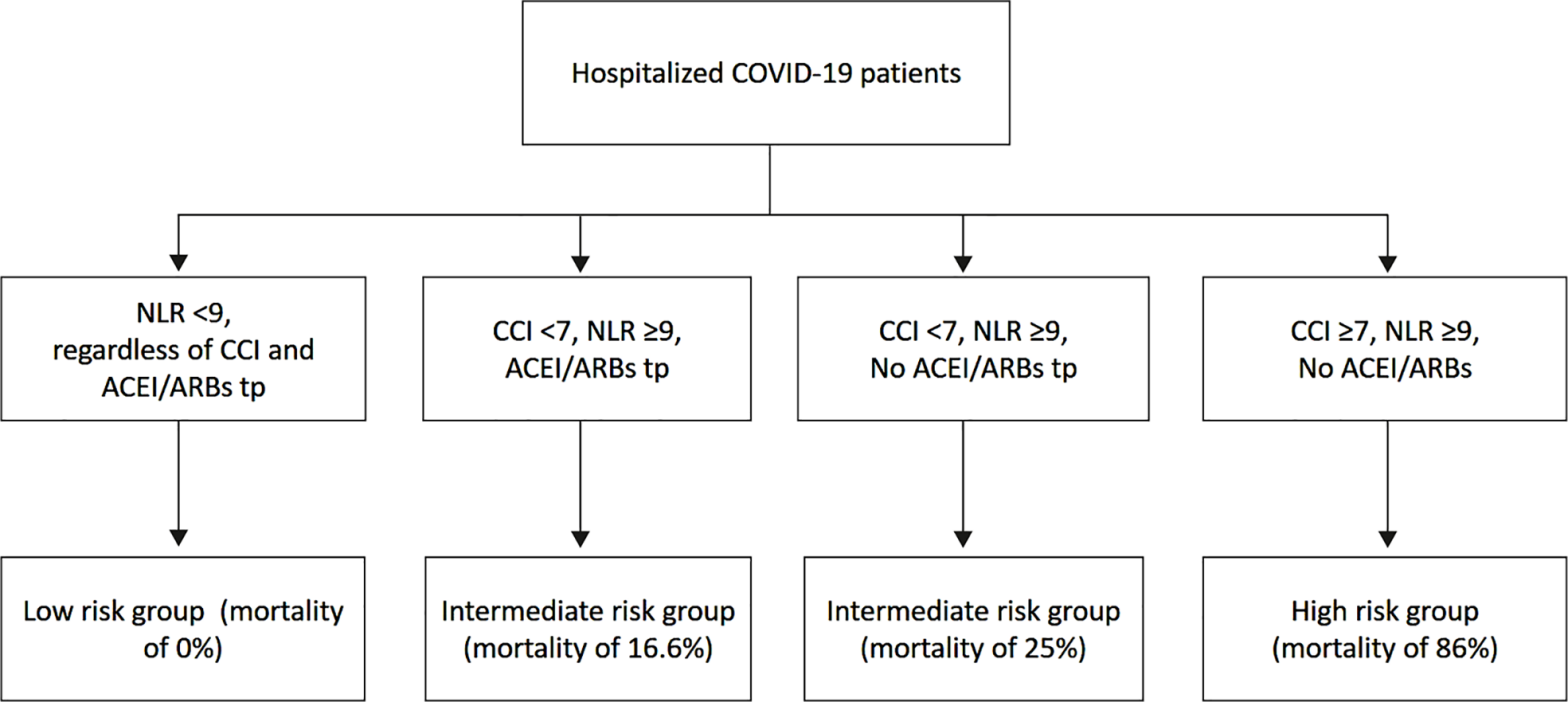
Chart of risk stratification of in-hospital mortality for our series of hospitalized COVID-19 patients by using CCI, NLR and ACEI/ARBs therapy. ACEI, angiotensin-converting-enzyme inhibitors; ARBs, angiotensin receptor blockers; CCI, Charlson comorbidity index; NLR, neutrophil-to-lymphocyte ratio.

A chart of risk stratification of in-hospital mortality drawn for our series of hospitalized COVID-19 patients by using CCI, NLR and ACEI/ARBs therapy, is illustrated in **Figure 4**. The mortality risk for patients with CCI ≥7, NLR ≥9 and without ACEI/ARBs therapy was very high (86%); for patients with CCI <7, NLR ≥9, with (16.6%) or without (25%) ACEI/ARBs therapy was intermediate; for patients with CCI <7, NLR <9 and with ACEI/ARBs therapy was of 0%.

## Discussion

The present study carried out on a retrospective cohort of 74 hospitalized COVID-19 patients during the Omicron dominant period revealed that: 1) the in-hospital mortality rate was 27% for the overall sample (20 of 74 patients); 2) compared to patients who were discharged alive, those who died during hospitalization were significantly older, had significantly greater prevalence of incomplete anti-COVID-19 vaccination, showed significantly higher comorbidity burden (as expressed by CCI), increased inflammatory biomarkers (especially WBCs and NLR), marked radiographic and echographic congestive signs, and were generally underprescribed with cardioprotective drugs (especially ACEI/ARBs) at hospital admission; 3) the baseline CCI and NLR were strongly correlated each other in the whole study group; 4) the main independent predictors of “in-hospital mortality” were the CCI, the baseline NLR and the undertreatment with ACEI/ARBs at hospital admission; notably, a CCI score ≥7 and a NLR ≥9 were the best cut-off values for predicting the outcome.

The overall in-hospital mortality rate detected in our series of COVID-19 patients was higher than that observed in previous studies which included younger patients (16, 17) and similar to that observed in other studies which enrolled geriatric patients with several comorbid conditions (18, 19).

During the last two years, a great number of studies reported that advanced age, male sex and multiple comorbidities, such as diabetes, cardiovascular, cerebrovascular, and respiratory diseases, are independent risk factors of mortality for COVID-19 patients (20–30). On the other hand, other studies showed that comorbidities were not effective predictors of mortality in these patients (31, 32). These different findings were likely related to different study designs and/or populations, or to the influence of confounding factors.

In the present study, to evaluate the influence of comorbidities on the patients’ outcome we employed the Charlson Comorbidity Index (CCI) score, a well-validated, simple and valid method for estimating risk of death from comorbid disease (11). It summarizes a number of comorbidities, each given a weighted integer from one to six depending on the severity of the morbidity. Consistent with previous studies (7, 33–36), we demonstrated that a higher CCI is strongly associated with increased mortality in COVID-19 patients. In our findings, the ROC curve analysis showed that a CCI threshold ≥7 yielded the best cut-off point for predicting mortality in COVID-19 patients.

Our results also revealed that various inflammatory biomarkers, such as WBCs, NLR, CRP, and procalcitonin, were elevated in the great majority of hospitalized COVID-19 patients. However, logistic regression analysis highlighted that, among these inflammatory biomarkers, only the NLR was independently associated with the primary endpoint in our retrospective cohort of patients.

The NLR, easily calculated from a routinely blood test by dividing absolute neutrophil count by absolute lymphocyte count, is a biomarker of systemic inflammation (37). The high NLR results from increased neutrophil count and decreased lymphocyte count. It’s related to the inflammatory response which stimulates the production of neutrophils and speed up the apoptosis of lymphocytes (38).

NLR has been widely used for predicting in-hospital mortality not only in infectious diseases but also in malignancy, cardiovascular diseases, intracerebral hemorrhage, polymyositis and dermatomyostis (39–43).

Concerning COVID-19 patients, several studies demonstrated that higher NLR levels on admission were associated with severe COVID-19 and mortality (16, 44–46).

In determining the optimal cut-off value of NLR for predicting outcome in COVID-19 patients, NLR values ranging from 3.3 to 5.9 predicted severity in some studies (47, 48), whereas higher NLR values ranging from 7.9 and 11.8 predicted mortality in other studies (49, 50). In our findings, a cut-off value of NLR ≥9 was the best cut-off value for predicting mortality.

The increase in serum levels of NLR indicates an imbalance in the inflammatory response where inflammatory factors related to viral infection, such as interleukin-6, interleukin-8, and granulocyte colony-stimulating factor, stimulate neutrophil production (47) and, in contrast, systemic inflammation accelerates lymphocyte apoptosis, depresses cellular immunity, decreases CD4+, and increases CD8+ suppressor T-lymphocytes (51, 52).

Bacterial co-infections due to low immune functions would be another possible reason for explaining the increased levels of NLR and other inflammatory biomarkers, such as CRP and procalcitonin, in COVID-19 patients with severe disease manifestation.

High levels of NLR may also be related to different combinations of comorbidities, as detected in our study population. Interestingly, we observed a strong correlation between NLR and CCI in hospitalized COVID-19 patients, suggesting that aging and comorbidities sinergically contribute to a higher basal proinflammatory status (53). It’s known that, at baseline state, the lungs of old individuals show increase in levels of complement and surfactant proteins and pro-inflammatory cytokines (54, 55). These factors can contribute to both pulmonary and systemic exacerbated inflammatory response in older individuals and seem to play a role in increasing susceptibility to respiratory infections (53).

Another important prognostic indicator assessed by our logistic regression analysis was the undertreatment with ACEI/ARBs at hospital admission in COVID-19 patients. Indeed, the mortality rate was significantly lower in patients chronically treated with ACEI/ARBs in comparison to patients not treated with ACEI or ARBs. Our findings would support the assumption that the up-regulation of angiotensin-converting enzyme (ACE)-2, a carboxypeptidase that cleaves angiotensin II into angiotensin- (1–7, 56, 57), induced by both ACEIs (58–60) and ARBs (61), could be potentially useful in the clinical course of SARS-CoV-2-infected patients, due to the cardiovascular protection elicited by the increased activity of angiotensin (1–7), thereby attenuating angiotensin II effects on vasoconstriction and sodium retention (57, 59). Therefore, our results are in alignment with previous studies that demonstrated a significantly lower mortality rate in hospitalized COVID-19 patients treated with ACEI/ARB therapy (62–67).

A possible explanation for the undertreatment with cardioprotective drug, especially ACEI/ARBs and beta blockers, observed in our cohort of COVID-19 patients at hospital admission, might be ascribable to the increased prevalence of comorbid conditions such as CKD and COPD; we believe that the clinicians were reluctant to prescribe ACEI/ARBs to older patients with impaired renal function and increased risk of hyperkalemia and/or to administer beta blockers to patients with COPD and increased risk of bronchospasm, hypotension or bradicardia.

To sum up, the results of the present study may help the clinicians to identify, among the hospitalized patients with COVID-19 infection, those with increased risk of in-hospital mortality. Those patients who are found with CCI ≥9, NLR ≥7 and who are not treated with ACEI/ARBs at hospital admission have a significantly increased risk of in-hospital mortality during COVID-19 infection. In other terms, those patients who are elderly, frail and with multiple comorbidities, who are found with increased inflammatory biomarkers at hospital admission, and who are not adequately treated with cardioprotective drugs, should be considered high-risk patients with more severe clinical presentation of SARS-CoV2 infection and significantly reduced survival probability. On the other hand, COVID-19 patients with CCI <9, NLR <7 and chronically treated with cardioprotective drugs have a significantly increased probability to be discharged alive from hospital.

Main limitation of the present study were the monocentric design of the study, its retrospective nature and the limited sample size of hospitalized COVID-19 patients analyzed. In the present study, Omicron was not confirmed through whole genome sequencing of SARS-CoV-2, which is the gold standard for genomic surveillance (68), not available at our Institution. However, the cases of COVID-19 patients included in this retrospective analysis were primarily attributed to Omicron, based on the global epidemiological temporal updates. In addition, blood tests did not include inflammatory biomarkers such as IL-6 and TNF-alfa, not assessed for the routinely evaluation of COVID-19 patients at our Center. Although a general undertreatment with cardioprotective drugs at hospital admission might have been the main factor responsible for a poor prognosis in our study group, the logistic regression analysis highlighted the independent prognostic role of ACEI/ARBs, only. An external validation cohort and adequately powered, prospective studies are needed to strengthen our results. A further study could be performed to investigate the composite of mortality and rehospitalization for all-causes in the same study population over a 6 and/or 12 months follow-up and/or to evaluate if the introduction and/or uptitration of cardioprotective drugs might improve the prognosis of these patients.

## Conclusions

The hospitalized COVID-19 patients included in this retrospective analysis showed a 27% of in-hospital mortality rate.

A high comorbidity burden, high levels of NLR and the undertreatment with ACEI/ARBs at hospital admission were the main independent prognostic indicators of in-hospital mortality in our series of patients.

The risk stratification of COVID-19 patients at hospital admission would help the clinicians to take care of the high-risk patients and reduce the mortality.

## Data availability statement

The datasets presented in this study can be found in online repositories. The names of the repository/repositories and accession number(s) can be found below: https://zenodo.org/record/7015021.

## Ethics statement

The studies involving human participants were reviewed and approved by Comitato Etico Indipendente IRCCS MultiMedica. Written informed consent for participation was not required for this study in accordance with the national legislation and the institutional requirements.

## Authors contributions

AS, MR, RC, DE, and AC, conceptualization, data curation, investigation, methodology, software, visualization, and writing—original draft. AA and DN, data curation, methodology, writing—review and editing. GN, ML, SH, conceptualization, supervision, validation, writing—review and editing. All authors contributed to the article and approved the submitted version.

## Funding

This research was supported by a grant from the Ministero della Salute COVID-2020-12371849 to DN and progetto RCR-2021-23671212. DN is also the recipient of a grant from Programmi di Ricerca Scientifica di Rilevante Interesse Nazionale (PRIN) Grant 2010 NECHBX_003 (to DN). Studies are partially funded by the Italian Ministry of Health Ricerca Corrente-IRCCS MultiMedica.

## Conflict of interest

The authors declare that the research was conducted in the absence of any commercial or financial relationships that could be construed as a potential conflict of interest.

## Publisher’s note

All claims expressed in this article are solely those of the authors and do not necessarily represent those of their affiliated organizations, or those of the publisher, the editors and the reviewers. Any product that may be evaluated in this article, or claim that may be made by its manufacturer, is not guaranteed or endorsed by the publisher.

## Abbreviations

2D, two-dimensional, ACEI, angiotensin-converting-enzyme inhibitors, ARBs, angiotensin receptor blockers, BMI, body mass index, BSA, body surface area, CAD, coronary artery disease, CCI, charlson comorbidity index, Cis, confidence intervals, CRP, C-reactive protein, CKD, chronic kidney disease, COPD, chronic obstructive pulmonary disease, COVID-19, Coronavirus disease 2019, CT, computed tomography, CXR, chest X-rays, ECG, electrocardiography, eGFR, estimated glomerular filtration rate, Hb, hemoglobin, HS, high-sensitivity, LVEF, left ventricular ejection fraction, NLR, neutrophil-to-lymphocyte ratio, NT-proBNP, N-terminal pro-B-type natriuretic peptide, ROC, receiver operating characteristic curve, RT-PCR, reverse-transcriptase polymerase chain reaction, SARS-CoV-2, severe acute respiratory syndrome coronavirus-2, SPAP, systolic pulmonary artery pressure, TIA, transient ischemic attack, TTE, transthoracic echocardiography, WBCs, white blood cells.
